# The *Drosophila* ribonucleoprotein Clueless is required for ribosome biogenesis *in vivo*

**DOI:** 10.1016/j.jbc.2024.107946

**Published:** 2024-10-30

**Authors:** Aditya Sen, Ambar Rodriguez-Martinez, Sara K. Young-Baird, Rachel T. Cox

**Affiliations:** 1 Department of Biochemistry and Molecular Biology, Uniformed Services University, Bethesda, Maryland, USA; 2 Henry M. Jackson Foundation, Rockville, Bethesda, USA

**Keywords:** ribosome, rRNA, ribosome biogenesis, mitochondria, nucleus, protein synthesis, Clu, *Drosophila*

## Abstract

As hubs of metabolism, mitochondria contribute critical processes to coordinate and optimize energy and intermediate metabolites. *Drosophila* Clueless (Clu) and vertebrate CLUH are ribonucleoproteins critical for supporting mitochondrial function; yet do so in multiple ways. Clu–CLUH bind mRNAs, and CLUH regulates mRNA localization and translation of mRNAs encoding proteins destined for mitochondrial import. In addition, Clu associates with ribosomal proteins and translation factors; yet whether it is required for fundamental ribosome function *in vivo* is not clear. In this study, we examine the Clu interactome and probe Clu’s requirement in ribosome biogenesis. We previously showed that Clu associates with ribosomal proteins. In this study, we extend these observations to show that *clu* null mutants display a significant decrease in overall protein synthesis. In addition, Clu associates with ribosomal proteins in an mRNA-independent manner, suggesting Clu’s core ribosomal function may be separate from its role in localizing and translating specific mRNAs. We find that Clu is present in the nucleus and associates with the rRNA processing protein fibrillarin but, surprisingly, that processed rRNA products are normal in the absence of Clu. Furthermore, Clu loss does not affect ribosomal protein levels but does result in a decrease in 40S and 60S ribosomal subunit abundance. Together, these results demonstrate that Clu is present in the nucleus and required for 40S and 60S biogenesis and global translation *in vivo*. These results highlight the multifaceted role of Clu in supporting cell function through regulation of mRNA encoding mitochondrial proteins and ribosome biogenesis.

*Drosophila* Clueless (Clu) is a member of the highly conserved Clustered (CLU) mitochondria superfamily of ribonucleoproteins that is required for mitochondrial function (1, 2, 3, 4). *clu* mutant flies have short lifespans, low ATP, mislocalized mitochondria, and are smaller compared to WT flies (1, 5). Both *Drosophila* Clu and yeast Clu1p bind mRNA, and CLUH predominantly binds mRNA encoding critical mitochondrial proteins although whether loss of CLUH decreases the level of CLUH-associated mRNAs is not clear (3, 6, 7, 8). Clu loss decreases mitochondrial protein abundance (3, 7, 9). Clu and CLUH associate with ribosomes at the mitochondrial outer membrane suggesting that Clu could be involved in cotranslational import of mitochondrial proteins, but whether Clu’s ribosomal association requires mRNA is unknown (6, 7, 10). In *Drosophila*, Clu forms large dynamic particles that are frequently associated with mitochondria that could potentially be sites of cotranslational import (11). An open question is whether Clu’s association with the ribosome is solely through its role as a ribonucleoprotein regulating mRNA stability, or whether it is also involved in ribosome function and regulation of protein synthesis.

Prior work from our group and others demonstrated that *Saccharomyces*, *Drosophila*, and *Arabidopsis* Clu homologs associate with translation initiation factors (7, 12, 13). We also previously demonstrated that Clu associates with ribosomal proteins from the large and small ribosomal subunit complexes (7). CLUH also associates with ribosomal proteins as shown by mass spectrometry (MS) (6). Furthermore, Clu sediments in the higher weight fractions containing polysomes, and this sedimentation shifts to lighter fractions upon ribosome disruption (7). Cluh/CLUH knockout or knockdown in mouse and human cell culture does not impact overall levels of protein synthesis (3, 6, 14). However, CLUH loss shifts the cell’s metabolic state in an mTORC1-dependent manner, a major regulator of protein synthesis (14, 15, 16, 17).

In this study, we sought to better understand Clu’s ribosomal association and how Clu loss affects ribosome function. Using polysome profiling, we found that *clu* null mutant flies have a greatly reduced polysome to monosome ratio, in contrast to the vertebrate cell culture studies. Furthermore, *clu* mutant larvae have little puromycin incorporation, corroborating our polysome profiling and suggesting a decrease in active protein synthesis. Clu binds ribosomal proteins in an mRNA-independent manner providing further support for a ribosome-specific role that does not require Clu’s mRNA association. Clu is highly abundant in the cytoplasm, but we find that Clu is also present in the nucleus and associates with the nucleolar methytransferase Fibrillarin (Fib), part of the small nucleolar RNA complex that processes rRNA. We show that decreased polysomes in c*lu* mutants are not because of defects in rRNA processing and that *clu* mutants have normal ribosomal protein levels. Instead, *clu* mutants display a reduction in 40S and 60S ribosomal subunit abundance. Overall, these data strongly support a role for Clu in regulating ribosome biogenesis and overall protein synthesis that is independent of its role modulating specific mRNA translation or stability.

## Results

### Clu is important for global protein synthesis *in vivo*

**Figure 1:**
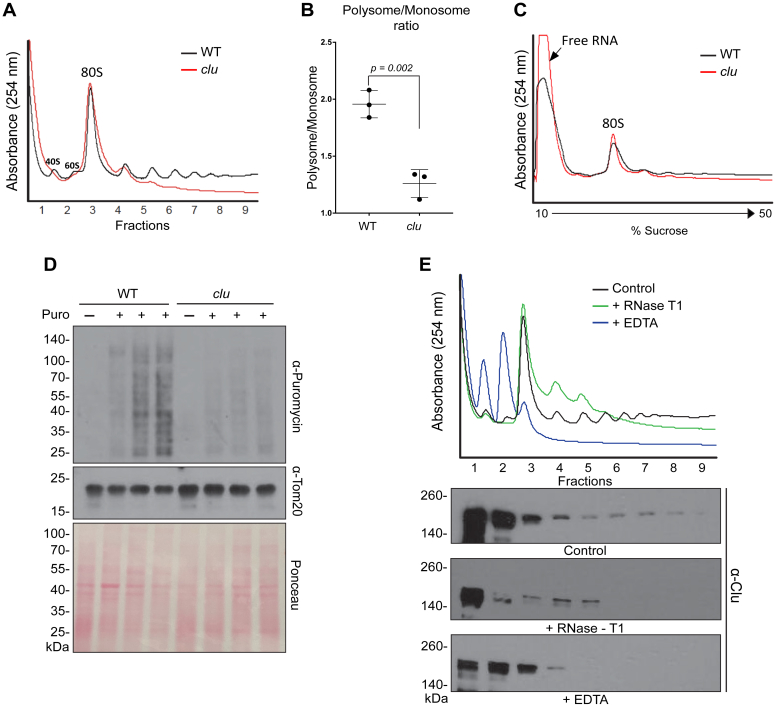
**Loss of Clu decreases overall levels of protein synthesis.***A*, polysome profiles of WT (*black trace*) and *clu* null mutant adults (*red trace*). *B*, comparing the area under the curve for polysomes *versus* monosomes indicates Clu loss greatly reduces the polysome–monosome ratio. *C*, the same polysome profiles from *A* with the trace zoomed out, extending the *y*-axis to visualize the free RNA peak. *D*, Western blots from WT and *clu* mutant larvae exposed to puromycin. Anti-Tom20 antibody and Ponceau are the loading controls. *E*, polysome profiles from WT adults that are untreated (control, *black trace*), treated with RNase T1 (*green trace*), or EDTA (*blue trace*). Western blots of the fractions indicate Clu is present in all fractions in the control. Error bars = SEM in *B*. Area under the curve (AUC) to analyze the polysome–monosome ratio was from three biological replicates and calculated using Microsoft Excel. Each *dot* represents an independent biological replicate. GraphPad Prism was used to make the graph. Unpaired *t* test in GraphPad Prism was used to calculate the significance.

We previously showed that *Drosophila* Clu associates with ribosomal proteins (7). However, the functional outcome of this association is unknown. To better understand how Clu affects ribosome dynamics, we performed polysome profiling using extract from *clu* null mutant (*clu* mutants) adult flies, which are protein nulls. These mutants develop through pupation and eclose into adult flies, but the adults are weak and small and only live four to five days (1, 5). Comparing *clu* mutant flies to their WT counterparts, we found that the *clu* mutation causes a decrease in polysome peak height compared with WT (Figs. 1*A*, S1*A*) and displays a statistically significant difference in the polysome to monosome ratio (Fig. 1*B*). Focusing on the free RNA peak near the top of the sucrose gradient, we found that the *clu* mutants displayed an increase in free RNA (Figs. 1*C*, S1*B*). As a control, we also performed polysome profiling on extract from *Superoxide Dismutase two* (*Sod2*) mutants, which have impaired mitochondrial function and less ATP than *clu* mutants (5, 7, 18). Our findings revealed that the loss of Sod2 results in a moderate reduction of polysomes (Fig. S1*C*) and a slight increase in free RNA (Fig. S1*D*) compared with WT but not as extreme as *clu* mutants. This suggests that low ATP is not wholly responsible for the decreased protein synthesis observed in *clu* mutants. Since rRNA is the most abundant RNA species, the increased free RNA peak suggests that there is an excess of unincorporated rRNA in *clu* mutants. As *clu* mutants show aberrant polysome profiles, we conducted a puromycin incorporation assay *ex vivo* to evaluate the active protein synthesis of these mutants. Puromycin is an aminonucleoside that mimics aminoacyl tRNA and upon incorporation blocks protein synthesis by causing premature termination of nascent polypeptide chains (19). Incubating *clu* mutant and WT larvae in puromycin-containing media, we observed that *clu* mutants incorporated substantially less puromycin compared with WT (Fig. 1*D*). We also assessed puromycin incorporation in cultured S2R+ cells and found that knock down of Clu did not impact protein synthesis in cultured cells (Fig. S1*E*). Overall, these data suggest that in contrast to mammalian cell culture, impaired Clu function in *Drosophila* causes a decrease in global protein synthesis, and this is not simply because of decreased ATP levels.

To determine where Clu localizes in the polysome profile, we isolated total protein from the sucrose gradient fractions and conducted Clu immunoblot analyses (Figs. 1*E*, S1*F*). Clu was most abundant in the 40S and 60S peaks but was also present in the polysome fractions in control samples treated with cycloheximide to stall elongating ribosomes (Fig. 1*E*, *black trace*, *upper blot*). To disrupt polysomes, we treated extracts with either EDTA or RNase T1. Treatment with RNase T1, a sequence-specific endoribonuclease, abolished the heavy polysomes while leaving the monosomic and light polysome peaks intact (Fig. 1*E*, *green trace*). There was a concomitant compaction of Clu to the free subunit, monosomic, and light polysome fractions (Fig. 1*E*, *middle blot*). Treating with EDTA that causes the 40S and 60S ribosomal subunits to dissociate resulted in a collapse of the polysome and 80S peaks and a corresponding increase in 40S and 60S subunit peak heights (Fig. 1*E*, *blue trace*). Furthermore, EDTA treatment caused a further sedimentation of Clu in the lighter fractions (Fig. 1*E*, *lower blot*). These results are consistent with our previous observations that Clu associates with mRNA and is present in heavier fractions on sucrose gradients (7).

### Clu associates with ribosomal proteins in an RNA-independent manner

**Figure 2:**
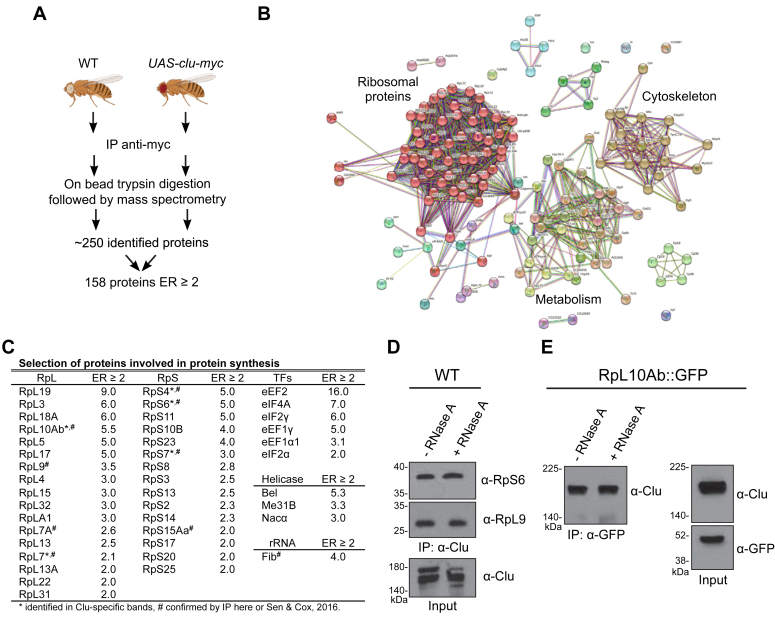
**Clu interacts with ribosomal proteins in an RNA-independent manner.***A*, schematic summarizing the immunoprecipitation (IP) and mass spectrometry experiment. The full list of identified proteins can be found in Table S1. *B*, STRING diagram summarizing the Clu-associated proteins identified from the IP experiment. Nodes represent proteins, and edges denote interactions. The color of each node represents the nature of interactions, for example, known or predicted interaction. A detailed description of nodes and edges can be found at STRING database (https://string-db.org/). *C*, the table listing proteins involved in protein synthesis identified from the mass spectrometry experiment. *Asterisks* indicate those ribosomal proteins also found in the mass spectrometry analysis of excised gel bands (Fig. S2). *Hashtags* indicate Clu-interacting candidates that were verified with IP. RpL, large ribosomal protein; RpS, small ribosomal protein; TF, translation factor. *D* and *E*, Western blots of IPs performed from extract isolated from WT (*D*) and RpL10Ab::GFP trap fly stock (*E*) using anti-Clu and anti-GFP antibodies, respectively, and treated with and without RNase A. ER, enrichment ratio.

To identify proteins that interact with Clu, we conducted Clu immunoprecipitation (IP) followed by MS. We used transgenic flies that ubiquitously overexpressed myc-tagged Clu (*UAS*-*clu*-*myc*) and immunoprecipitated using anti-myc antibody (Fig. 2*A*). We previously showed that ectopically expressing *UAS*-*clu* using the same ubiquitously expressing *daughterless* (*da)GAL4* driver rescues the *clu* mutant phenotypes, indicating that this construct is fully functional (7). Mass spectrometric analysis of the immunoprecipitate identified 242 proteins and showed 158 proteins with an enrichment ratio ≥2 or uniquely associated with Clu compared with WT control (Table S1). Clu was the most enriched protein identified (peptide-spectrum match = 90 in experiment and zero in control), supporting the specificity of the IP. Functional clustering analysis using STRING revealed three major classes: ribosomal proteins, proteins involved in the cytoskeletal network, and proteins involved in metabolism (Fig. 2*B*). Since Clu loss affects protein synthesis and we previously identified Clu as interacting with ribosomal proteins, we were particularly interested in the identified proteins involved in protein synthesis including ribosomal proteins and the rRNA processing enzyme Fib (Fig. 2*C*). Previously, we performed IP using anti-Clu antibody in WT flies and analyzed three unique Clu-associated bands *via* MS, which identified additional ribosomal proteins (Fig. S2, *A* and *B*, Table S2, (7)). To validate the MS data identifying ribosomal proteins, as well as extend our previous data showing Clu–ribosomal protein association, we examined Clu’s ability to associate with additional ribosomal proteins based on commercial antibody availability for antibodies that crossreact with *Drosophila* (7). Clu associates with RpS6, RpS15Aa, and RpL9 using IP from extract isolated from WT flies (Figs. 2*D*, S2, *C* and *D*). Clu also associates with RpL10Ab using IP from extract isolated from a fly stock expressing RpL10Ab fused with GFP at the endogenous locus, as we have previously shown (Fig. 2*E*, (20, 7)). Importantly, in fly extracts treated with RNase A, Clu retained its association with RpL10Ab, RpS6, and RpL9 (Fig. 2, *D* and *E*). These observations emphasize that while Clu is a ribonucleoprotein, it interacts with the ribosome in an mRNA-independent manner.

### Clu is in the nucleus and associates with fibrillarin

**Figure 3:**
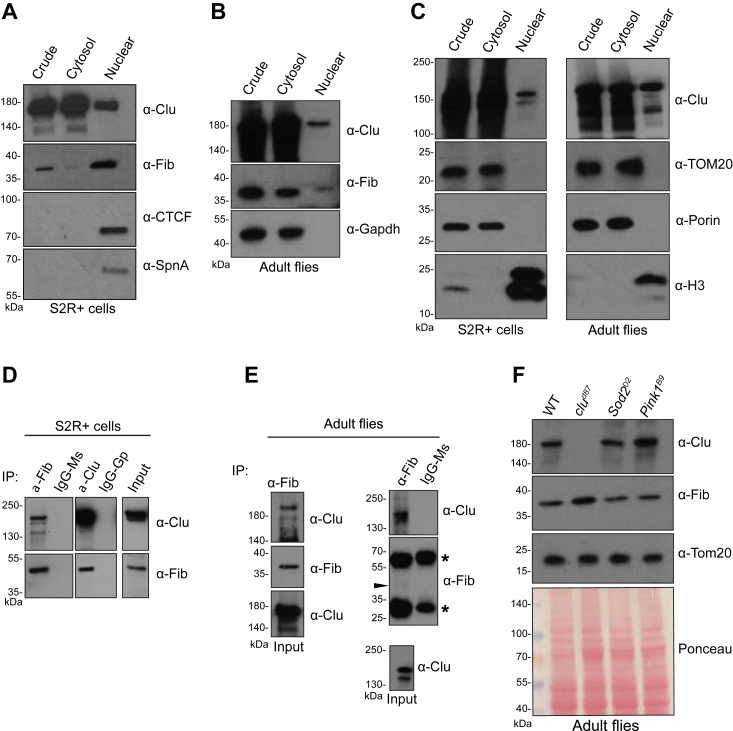
**Clu is found in the nucleus.***A* and *B*, Western blots of cytosolic and nuclear cell fractions. (CA) Fractionated S2R+ cell extract. The nuclear fraction is enriched 50x. Controls include CTCF (CCCTC-binding factor) and SpnA (DmRad51), which are located in the nucleus. *B*, fractionated extract from WT adult flies. Gapdh (Glyceraldehyde three-phosphate dehydrogenase) is a cytoplasmic control. *C*, fractionated S2R+ and adult fly extract. The nuclear fraction is enriched 50x. Antibodies against TOM20 and Porin indicate mitochondria are absent from the nuclear fraction. H3 (Histone H3) is predominantly in the nuclear fraction. *D* and *E*, Western blot analysis of Fibrillari (Fib)–Clu immunoprecipitations (IPs) from cultured S2R+ cells (*D*) and adult flies (*E*). Mouse IgG (IgG-Ms) or guinea pig IgG (IgG-GP) was used as a control IP for anti-Fib and anti-Clu, respectively. IgG-Ms was used as a control for anti-Fib IP. The *arrowhead* indicates the Fib band. *Asterisks* indicate nonspecific mouse IgG signal. For the Western blots in Fig 3*D*, we used a modified anti-Mouse Ig HRP to reduce the strength of the IgG heavy and light bands. *F*, Western blot analysis of Clu and Fibrillarin in adult flies. Tom20 and Ponceau are shown as loading controls. *Superoxide Dismutase two* (*Sod2*) and *PTEN Induced Kinase-1* (*Pink1*) are mutants that cause mitochondrial dysfunction and are present for additional controls.

Our polysome analysis showed that *clu* mutants display reduced polysome levels with a corresponding increase in the free RNA peak height (Figs. 1*C* and S1*B*). In addition, we identified Fib, a core member of the nuclear rRNA processing complex and a key protein in ribosome biogenesis, in the Clu IP-MS experiment (Fig. 2*C*, Table S1, (21)). Furthermore, a previous study showed that *CLUH* gene regulation correlates strongly with genes regulating ribosome biogenesis (3). Thus, even though Clu and its orthologs are known to be highly abundant in the cytosol, we wondered if Clu may also localize to the nucleus and play a role in ribosome biogenesis. To investigate Clu's cellular localization, we performed cell fractionation and found that Clu is present in the nucleus-enriched fractions from cultured S2R+ cells and adult flies (Fig. 3, *A*–*C*). To investigate whether Clu is involved in ribosome biogenesis, we verified the Clu–Fib interaction. Performing co-IP using anti-Fib antibody, we found that Clu associates with Fib in cultured S2R+ cells and adult flies (Fig. 3, *D* and *E*). Since Clu associated with Fib and overall translation is reduced in the *clu* mutants, we examined whether altered Fib levels could account for the reduced translation. We also examined extract from adult flies lacking Sod2 and PTEN-induced putative kinase one (Pink1). These two proteins are critical for mitochondrial function and served as controls for mitochondrial damage and stress (5, 7, 22, 23, 24). Western blots of extract showed that Fib levels are approximately the same in *clu* mutants as WT, thus reduced Fib levels cannot account for the reduced amount of polysomes (Fig. 3*F*). These results demonstrate a new subcellular localization for Clu in the nucleus and that Clu associates with the rRNA processing methylase Fib. While this newly identified subcellular localization could potentially explain Clu’s role in global protein synthesis, Clu loss did not affect Fib levels.

### 40S and 60S ribosomal subunits are reduced in the absence of clu

**Figure 4:**
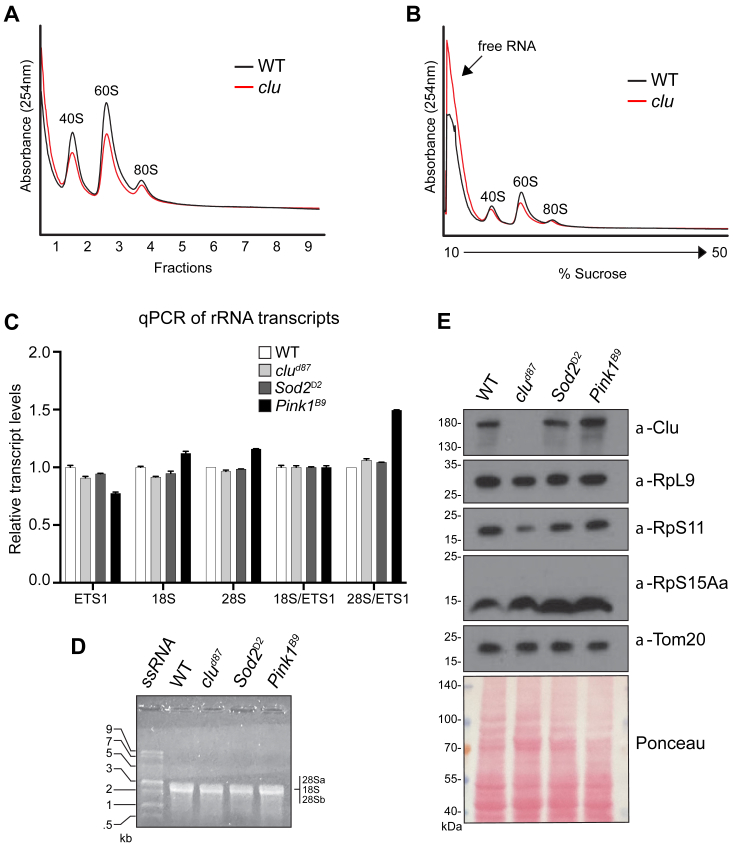
**The 40S and 60S ribosomal subunits are reduced with Clu loss.***A*, polysome profile of WT and *clu* null mutant fly extract in the presence of 10 mM EDTA and the absence of cycloheximide and MgCl_2_. *B*, the same polysome profiles from *A* with the trace zoomed out, extending the *y*-axis to visualize the free RNA peak. *C*, quantitative PCR analysis of rRNA processing. Steady-state levels of the 5′ external transcribed spacer (ETS) intermediate and 18S and 28S processed rRNA levels in the WT and *clu* null mutant are shown. *Superoxide Dismutase two* (*Sod2*) and *PTEN-induced putative kinase one* (*Pink1*) mutants that cause mitochondria dysfunction are presents as controls. *Bars* represent an average of three technical replicates. Error bars = standard error of mean. The graph was created using GraphPad Prism. *D*, RNA gel electrophoresis. Total RNA from adult flies run on a nondenaturing 1% TBE-agarose gel. The 28Sa/18S/28Sb rRNA product bands are indicated. These three products have very similar sizes (Fig. S3*B*, (45)). *E*, Western blot analysis of ribosomal protein RpL9, RpS11, and RpS15A levels in WT and *clu* null mutants are shown. *Superoxide Dismutase* two (*Sod2*) and *PTEN Induced Kinase-1* (*Pink1*) are mutants that cause mitochondrial dysfunction and are present for additional controls. Anti-Tom20 antibody and Ponceau are shown as loading controls.

Since a portion of Clu localizes to the nucleus and associates with Fib, it is possible that reduced global translation in the *clu* mutant causes deficits in ribosome biogenesis. To determine if ribosome biogenesis is disrupted, we measured the levels of the 40S and 60S subunits. We performed polysome profiling from fly extracts from *clu* mutant and WT flies in the presence of EDTA, which disrupts the 80S monosome, thus enhancing the 40S and 60S peaks. Our results showed that both 40S and 60S peaks were low in *clu* mutants compared with WT flies (Fig. 4*A*). As with our previous observation, *clu* mutants have increased free RNA compared with WT (Fig. 4*B*). One possibility for the reduction in the 40S and 60S ribosomal subunit levels is incorrect rRNA processing. To test this, we used quantitative PCR (qPCR), RT–PCR, and agarose gel analyses to monitor several rRNA species for changes to steady-state levels and junction processing. rRNA processing is a complex, multistep process (Fig. S3*A*). In the first step of rDNA transcription, an unprocessed polycistronic rRNA is synthesized by the RNA polymerase I complex and subsequently processed by snoRNPs composed of small nucleolar RNAs and several core nucleolar proteins that cleave the unprocessed rRNA in complex and well-coordinated steps resulting in several intermediate rRNA species (25). We designed primers to measure steady-state levels of two rRNAs, 28Sa and 18S, as well as the 5′ external transcribed spacer (ETS1) whose formation is required for 18S rRNA formation and is the longest noncoding region in the rRNA transcript (Fig. S3) (26). As with monitoring Fib levels previously, we also analyzed rRNA transcripts in *Sod2* and *Pink1* mutants to control for other types of mitochondrial stress. Our qPCR analysis revealed that the levels of ETS1, 18S, and 28Sa did not significantly change in *clu* mutants compared with WT (Fig. 4*C*). Furthermore, agarose gel analysis of total RNA confirmed that the levels of the 28Sa/18S/28Sb RNA band did not change between WT and *clu* mutant flies (Fig. 4*D*). In *Drosophila*, the 28S transcript is cleaved into two products, 28Sa and 28Sb, that have similar sizes (Fig. S3*B*, (27, 28)). We also performed RT–PCR and qPCR using primers flanking junctions and found no change in the amount of product between WT and *clu* mutants (Fig. S4). As there was no decrease of rRNA products or fibrillarin in *clu* mutants, we investigated the levels of a subset of ribosomal proteins using commercially available antibodies that crossreact with *Drosophila*. Western blot analysis showed that there were no apparent major changes in the levels of RpL9, RpS6, RpS11, and RpS15Aa in *clu* mutants or in *Sod2* and *Pink1* mutants (Fig. 4*E*). In conclusion, these data highly implicate Clu as an important regulator of ribosome biogenesis and protein synthesis.

## Discussion

### Clu’s role in translation apparatus activity

The CLU mitochondria superfamily is a critical group of ribonucleoproteins that support mitochondrial function. However, it is not entirely clear whether Clu’s primary cellular effects are through regulating the translation of specific mRNAs, global translation impacts, or both, and how changes to these pathways affect mitochondrial function. Clustered A (CluA), the *Dictyostelium* founding member of the CLU superfamily, was originally identified as essential for normal mitochondrial cellular distribution (29). This mitochondrial clustering phenotype is observed in all species with CLU-superfamily loss (1, 2, 4, 14). Shortly after this initial observation, Clu1p was identified in yeast as a nonessential component of the eukaryotic initiation factor three (eIF3) complex (13). We confirmed that *Drosophila* Clu associates with eIF3g and demonstrated that Clu associates with several ribosomal proteins (7). In addition, we tested one ribosomal protein, RpL7A, and found it associates with Clu in the mitochondrial fraction, suggesting Clu can bind to ribosomes located at the mitochondrial outer membrane (7). The *Arabidopsis* homolog FMT also associates with cytosolic ribosomes at the mitochondrial outer membrane and with translation factors (10, 12). Furthermore, a recent study combining proximity-labeling and RNA-IP has shown that CLUH binds mRNAs and their protein product at mitochondrial outer membranes (6). Clu is a ribonucleoprotein that binds mRNA as shown by RNA-IP, and CLUH binds mRNAs that predominantly encode mitochondrial proteins as demonstrated by crosslinking and IP and RNA-IP followed by next-generation sequencing (3, 7). CLUH may regulate mRNA stability, although this is not entirely clear as there are conflicting results (6, 8, 14).

In this study, we found that Clu associates with ribosomal proteins in an mRNA-independent manner, suggesting that mRNA binding may not be necessary for its possible role in regulating translation. Furthermore, *clu* null mutants display a global reduction in protein synthesis, as measured by polysome profiling and puromycin incorporation, which would explain why adult mutant flies are smaller relative to WT (1). We found decreased puromycin incorporation only occurred *in vivo* in *clu* mutant larvae. RNAi knockdown in cultured S2R+ cells did not show any difference, consistent with another study (3). This could be due to differences in the systems and cellular conditions (*in vivo versus* cell culture) or the residual amount of Clu left in the cells after RNAi treatment may be sufficient to support global translation. Clu is likely not a core component of the ribosome as it is a large protein that is not present in ribosome structural studies (30, 31, 32). Furthermore, fly mutants in ribosomal proteins are haploinsufficient and display well-characterized phenotypes including delayed development, sterility, short thin bristles, and other deformities (33, 34, 35). Homozygous null *clu* mutants display some of these phenotypes, such as delayed development, sterility, and reduced size but do not have thin bristles or other deformities, and *clu* is not haploinsufficient (1).

### The implications of clu subcellular localization for ribosome biogenesis

Clu is an abundant protein in the cytoplasm. We and others have shown that CLU superfamily members form dynamic particles in the cytoplasm that are often associated with mitochondria (1, 3, 11, 12). In *Drosophila*, Clu particles are unique and sensitive to stress, dispersing in response to oxidative and nutritional stress, as well as stress caused by mutations in genes required for mitochondrial function (11, 36). Whether Clu particles contain ribosomes has been difficult to address since the particles are challenging to isolate and ribosomes are highly abundant in the cytosol. How Clu particle function relates to Clu molecular function is unclear. Using a systems biology approach, the *CLUH* gene regulatory network was found to be highly correlated with genes involved in the regulation of ribosome biogenesis (3). We and others also find several ribosome biogenesis proteins in our co-IP MS experiments and demonstrate here that Clu is found in nuclear fractions and associates with Fib by co-IP. Clu’s nuclear localization may rely on piggybacking with ribosomal proteins since many ribosomal proteins shuttle in and out of the nucleus (37, 38). Clu’s association with Fib is likely indirect since Fib (Nop1 in yeast) genetically and physically interacts with many ribosomal proteins (39, 40). The *clu* mutant polysome profiles showed a substantial decrease in polysome peak height with a corresponding increase in the amount of free RNA. Since rRNA makes up the bulk of cellular RNA, and we did not identify increased rRNA intermediates, this suggests that there is excess rRNA that has not been incorporated into ribosomes. Supporting this hypothesis, the 40S and 60S peaks are decreased with Clu loss. We did not analyze the kinetics of rRNA processing; however, since our analysis of multiple rRNA species showed that levels of intermediates and final products are not reduced, this indicates that the decreased polysomes are not due to a reduction in rRNA products. In addition, levels of several ribosomal proteins in the *clu* mutant are similar to WT, suggesting that a lower concentration of ribosomal proteins does not explain the polysome decrease. These data support that Clu is somehow involved in assembly of the subunits. Fib is a highly conserved methyltransferase that methylates over 100 sites on rRNA during the initial steps of processing (41). It is involved in pre-rRNA cleavage, rRNA methylation, and ribosome assembly. We cannot rule out that Clu’s loss affects methylation activity and that this is responsible for increased free RNA and decreased 40S and 60S levels. However, since several rRNA species were at normal levels in the absence of Clu, this does not appear likely.

Ribosome biogenesis is an energy intensive process, requiring ATP/GTP-dependent enzymes and kinases for many steps (42). A simple explanation for decreased 40S and 60S subunits in *clu* mutants is that there is an insufficient amount of available ATP. *clu* mutants live only four to seven days, suffer from oxidative damage, and decreased ATP and ultrastructurally, have damaged looking mitochondria (1, 5). For our analysis, we used mutant flies that were 1 to 2 days old to ensure they were optimally healthy. At this age, the flies have approximately 50% the normal amount of ATP (5). To control for reduced ATP causing decreased polysomes and protein synthesis, we also examined extract from *Sod2* mutants. Sod2 is mitochondrially localized (43). *Sod2* mutants have oxidative damage and live 24 h (5, 18). *Sod2* mutants have only 20% of WT ATP levels compared with *clu* mutants, which have 50% WT levels, yet *Sod2* mutant extract showed an intermediate level of free RNA and amount of polysomes (Fig. S1, *C* and *D*, (5, 36)). Therefore, we believe that the ribosomal biogenesis defects that we observe could only partially be attributed to decreased ATP, especially considering that we demonstrated Clu is found in the nucleus.

### Outstanding questions and future directions

One challenge to understand Clu’s molecular roles in the cell is because Clu is a cytoplasmic protein that is involved in many different processes. Thus, which process, or processes, is most critical has been difficult to parse. And the hierarchy of importance with respect to pathway could change depending on the cell type. CLUH is clearly involved in metabolism and is affected by the mTORC1 pathway, and metabolic state greatly affects the subcellular localization of Clu. In addition, *clu* mutants have substantially reduced mitoribosomal proteins, a phenotype shared with *Pink1* mutants. As mitochondrial defects cause general stress through the integrated stress response, Clu’s cellular role could be at the first onset of stress, but once stress overwhelms the system and causes mitochondrial damage, Clu may shift to a different pathway. Thus, Clu may link to translation through multiple pathways. Determining how and when each pathway is used will be critical for understanding this highly conserved protein family.

## Experimental procedures

### *Drosophila* stocks and cell lines

The following fly stocks were used for experiments: for Fig. 2*A*, WT was *y, w*. For all other experiments, WT was *w*^*1118*^. *UASp*-*Clu*-*myc*, *clueless*^*d08713*^/*CyO Act GFP* (1), *Sod2*^*Δ2*^/*CyO Act GFP*, *Pink1*^*B9*^/*FM7*i (23), and *daughterless-Gal4*/*TM3* were obtained from the Bloomington *Drosophila* Stock Center. Flies were reared on standard cornmeal fly media at 22 or 25 °C. Full length *clu* was subcloned into a pENTR/D-TOPO vector from complementray DNA (cDNA) clone (RH51925) using the following primers: CACCATGGCGCTTGAAACGGAGGC (forward) and ACTTGAAGTCGCCTCAGTGGCTGC (reverse) using the pENTR/D-TOPO cloning kit (Thermo Fisher Scientific). The myc tag was added by swapping the Entry clone into the pPWM Gateway vector (The *Drosophila* Gateway Collection, Carnegie Institution of Washington/Addgene) using Gateway LR Clonase II kit (Thermo Fisher Scientific). *Drosophila* S2R+ cell line was obtained from *Drosophila* Genomics Resource Center (DGRC stock 150; https://dgrc.bio.indiana.edu//stock/150; Research Resource Identifier: CVCL_Z831 (44)). S2R+ cells were cultured in Schneider’s *Drosophila* Medium (catalog no.: 21720024; Thermo Fisher Scientific) supplemented with 10% heat-inactivated fetal bovine serum.

### IP and MS

IP was performed from WT and Clu-myc-tagged flies using anti-myc antibody (catalog no.: 05-429; MilliporeSigma) and Protein A/G magnetic beads (catalog no.: 88802; Thermo Fisher Scientific). After washing the beads three times with IP buffer (20 mM Hepes, pH 7.4; 50 mM KCl, 0.02% Triton X-100, 1% NP-40 [sub], 1 mM EDTA, 0.5 mM EGTA, and 5% glycerol), on-bead trypsin digestion was performed. MS analysis was performed commercially by Poochon Scientific. The digested peptide mixture was then concentrated and desalted using C18 Zip-tip and reconstituted in 0.1% formic acid. The LC/MS/MS analysis of samples were carried out using a Thermo Scientific Orbitrap Exploris 240 Mass Spectrometer and a Thermo Dionex UltiMate 3000 RSLCnano System. Raw data files were searched against *Drosophila melanogaster* sequence database (3766 entries) obtained from the National Center for Biotechnology Information website using the Proteome Discoverer 1.4 software (Thermo Fisher Scientific) based on the SEQUEST algorithm. The minimum peptide length was specified to be five amino acids. The precursor mass tolerance was set to 15 ppm, whereas fragment mass tolerance was set to 0.05 Da. The maximum false peptide discovery rate was specified as 0.01. The resulting report contains all assembled proteins with peptide sequences and peptide spectrum match counts. Protein identification from excised gel bands was carried out commercially using the NanoLC–ESI–MS/MS peptide sequencing technology. The process involved cleaning and digesting each protein sample in-gel with sequencing-grade modified trypsin. The resulting mixture of peptides was analyzed using an LC–ESI–MS/MS system, where a HPLC with a 75-μm inner diameter reverse-phase C18 column was on-line coupled with an ion trap mass spectrometer. The data obtained through MS were used to search the most recent nonredundant protein database from the National Center for Biotechnology Information.

### Quantitative PCR

qPCR was performed as described previously (9). In short, total RNAs were isolated from WT and mutant adult flies using a Direct-zol RNA MiniPrep Plus Kit (catalog no.: R2070; Zymo Research) as per the manufacturer’s recommended protocol. One microgram of total RNA was reverse transcribed using a High-Capacity cDNA Reverse Transcription Kit (catalg no.: 4368814; Thermo Fisher Scientific) in a 20 μl reaction. cDNA was later diluted appropriately. For gene expression analysis, qPCR was performed using Luna Universal qPCR Master Mix (catalog no.: M3003L; New England Biolab) in a 20 μl reaction with 20 ng of cDNA and respective primer pairs specific to ETS1, 18S, 28S, and ACT5C (endogenous control). Fold changes were measured based on ddCt values compared with the endogenous transcript ACT5C. ddCt values were converted to 2ˆ[ddCt] to better represent the exponential nature of PCR. The average of three technical replicate 2ˆ[ddCt] values for each sample was plotted in bar graphs in GraphPad Prism (GraphPad Software, Inc). The SEM and *p* values (unpaired *t* test) were calculated using GraphPad Prism.

### Polysome profiling

About 50 mg of frozen adult flies were homogenized using a 1 ml glass homogenizer (Wheaton) with ten strokes each by loose and tight pestle in 1 ml of complete polysome lysis buffer (20 mM Tris–Cl, pH 7.5, 100 mM NaCl, 0.4% NP-40, 10 mM MgCl_2_, 50 μg/ml cycloheximide, and one cOmplete EDTA-free protease inhibitor cocktail tablet [catalog no.: 4693132001; Roche/MilliporeSigma] per 50 ml buffer). To prepare the lysis buffer with EDTA, the complete polysome lysis buffer was supplemented with 10 mM EDTA and did not include MgCl_2_ or cycloheximide. The homogenized sample was spun twice at 4 °C for 5 min each at 700*g* to remove unbroken cells and cell debris. The lysate was then passed 10 times through a 25G needle. After that, the lysate was incubated on ice for 10 min on ice and then spun at 15,000*g* for 10 min. The supernatant was carefully removed to a fresh tube, and the total RNA was measured by Nanodrop (Thermo Fisher Scientific). An equal amount of extract was placed on top of a 10 ml premade continuous sucrose gradient (10–50%). It was then centrifuged at 40,000*g* on a Beckman SW41Ti rotor (Beckman Coulter Life Sciences) for 2 h at 4 °C. For polysome profiling, the gradients were fractionated and monitored at 254 nm using a BioComp Gradient Fractionator (BioComp Instruments). The fractions were immediately stored at −80 °C until further use. The polysome–monosome ratio was calculated using Microsoft Excel to calculate the area under the curve and then divided the area calculated for the polysomes by the area calculated for the monosomes.

### Extraction and precipitation of proteins from sucrose fractions

To prepare the protein sample from sucrose fractions, 150 μl of the fraction was transferred into a fresh tube. Subsequently, 600 μl of methanol was added and mixed by inverting the tube 10 times, followed by the addition of 150 μl of chloroform, which was vortexed for 3 to 5 s. Next, 450 μl of H_2_O was added to the mixture and vortexed again for 3 to 5 s. The resulting mixture was immediately centrifuged at 16,000*g* for 5 min at room temperature, and the upper aqueous layer was carefully removed without disturbing the white substance at the interface. To the remaining mixture, 650 μl of methanol was added and mixed by inverting the tube five times, followed by centrifugation at 16,000*g* for 5 min at room temperature, resulting in the formation of a white precipitate at the bottom of the tube. The liquid was aspirated without disturbing the pellet, and the pellet was dried by keeping the cap open for 30 min at room temperature. Finally, 80 μl of 1x SDS sample buffer (50 mM Tris–Cl, pH 6.8, 2% SDS, and 0.1% bromophenol blue) was added to the tube, which was then boiled for 8 min before analysis on gel.

### Fractionation and Western blots

To isolate nuclear-enriched fractions, S2R+ cells were first washed once with chilled 1X PBS (137 mM NaCl, 2.7 mM KCL, 10 mM Na_2_HPO_4_, and 1.8 mM KH_2_PO_4_) and then suspended in an appropriate volume of 1X complete hypotonic buffer (20 mM Tris–Cl, pH 7.5, 10 mM NaCl, 3 mM MgCl_2_, 0.5X Protease Inhibitor Cocktail [1 cOmplete EDTA-free protease inhibitor cocktail tablet (catalog no.: 4693132001; Roche/MilliporeSigma] per 100 ml buffer). Similarly, adult flies were ground in 1X complete hypotonic buffer in an Eppendorf tube using a blue pestle. The cell and fly extracts were then incubated on ice for 10 min. After the incubation, 1/20 volume of 10% NP-40 was added to the extract and vortexed for 10 s at high settings. About 0.5% NP-40 can completely disrupt mitochondrial membranes leaving the nuclear membrane intact. The extracts were then centrifuged at 300*g* for 5 min to remove unbroken cells. The supernatant was recentrifuged at 1200*g* for 10 min. The cytosolic supernatant was collected in a new tube, and the nuclear pellet was washed twice in 1X hypotonic solution. To extract the nuclear protein, the pellets were resuspended in 1x SDS sample buffer and boiled for 5 min. All prepared samples were kept frozen until further use. Western blotting was performed as previously described (5, 36). In short, protein samples from adult flies were extracted in a 1X SDS sample buffer. Samples were run on 4 to 15% TGX gels (catalog no.: 4561086; Bio-Rad Laboratories) and transferred on to a nitrocellulose membrane (catalog no.: 88018; Thermo Fisher Scientific) using a Trans-blot semidry apparatus (catalog no.: 1073940; Bio-Rad Laboratories). The membrane was incubated in Ponceau stain (catalog no.: P7170; MilliporeSigma) for 3 to 5 min and then imaged. After blocking in 5% dried milk, blots were probed against appropriate primary and horseradish peroxidase–conjugated secondary antibodies. For the Western blots in Fig 3*D*, we used Mouse TrueBlot ULTRA: Anti-Mouse Ig horseradish peroxidase (catalog no.: ABIN1589977; Antibodies Online) to reduce the strength of the IgG heavy and light bands. The blots were then exposed to SuperSignal West Pico PLUS Chemiluminescent Substrate (catalog no.: 34580; Thermo Fisher Scientific) and detected on X-films developed using an automated X-ray film processor (PROTEC GmbH & Co KG). Images from developed X-rays were captured using a color scanner, saved as JPGs, and converted to gray scale images using Photoshop. No other manipulation occurred. The following antibodies were used: anti-Clu (1:15,000 dilution) (1), anti-TOM20 (1:2000 dilution; Santa Cruz Biotechnology), anti-Fib (1:5000 dilution; catalog no.: ab5821; Abcam), anti-CTCF (1:2000 dilution, catalog no.: ab70303; Abcam), anti-GAPDH (1:3000 dilution, catalog no.: Ls-C19379; LSBio Shirley), anti-RpL9 (1:3000 dilution, catalog no.: sc-100828; Santa Cruz Biotechnology), anti-RpS6 (1:5000 dilution, catalog no.: sc-74459; Santa Cruz Biotechnology), anti-RpS11 (1:3000 dilution; catalog no.: A303-937A-M; Bethyl Laboratories), anti-RpS15Aa (1:3000 dilution; catalog no.: A304-990A-M; Bethyl Laboratories), anti-myc (1:5000 dilution; catalog no.: 05-429; MilliporeSigma), anti-Puromycin (1:500 dilution, catalog no.: PMY-2A4-s; Developmental Studies Hybridoma Bank).

### Puromycin assay

Six third instar larvae were inverted inside out in Grace's media using fine forceps and then transferred to an Eppendorf tube containing Grace's media mixed with puromycin (catalog no.: A11138-03; Thermo Fisher Scientific) at a final concentration of 10 μg/ml. The larvae were incubated for 40 min at room temperature on a nutator. After the incubation, the media were removed, and the sample was washed twice for ten min with chilled 1X PBS. The samples were then snap-frozen in dry ice and stored at −80 °C until further use.

### RNA gel analysis

Total RNA was extracted from adult flies using a Direct-zol RNA MiniPrep Plus Kit (catalog no.: R2070; Zymo Research). The RNA quality was evaluated using a Qubit 4 fluorometer (Thermo Fisher Scientific), and the concentration was measured using a Nanodrop (Thermo Fisher Scientific). To analyze the RNA species, 1 μg of RNA (diluted in 1X RNA dye from New England Biolabs) was loaded onto a 1% denaturing agarose gel, which was prepared in 1X Mops buffer (Fisher Scientific) containing 18% formaldehyde and 1X DNA safe Red Dye from Sigma (catalog no.: SCT123; MilliporeSigma). The gel was run at 50V for 40 to 50 min, or until the front dye front moved down two-thirds of the gel. Finally, the gel was visualized under a UV transilluminator.

## Data availability

All data are contained within the article.

## Supporting information

This article contains supporting information (36).

## Conflict of interest

The authors declare that they have no conflicts of interest with the contents of this article.
