# Supplementary material for: The Drosophila ribonucleoprotein Clueless is required for ribosome biogenesis in vivo
Source: J Biol Chem. 2024 Oct 30;300(12):107946. doi: 10.1016/j.jbc.2024.107946 (PMC11625335; doi:10.1016/j.jbc.2024.107946)
Supplement: Figure S1 [file mmc1.pdf]

Fig. S1 Replicates for the effect of Clu loss on translation

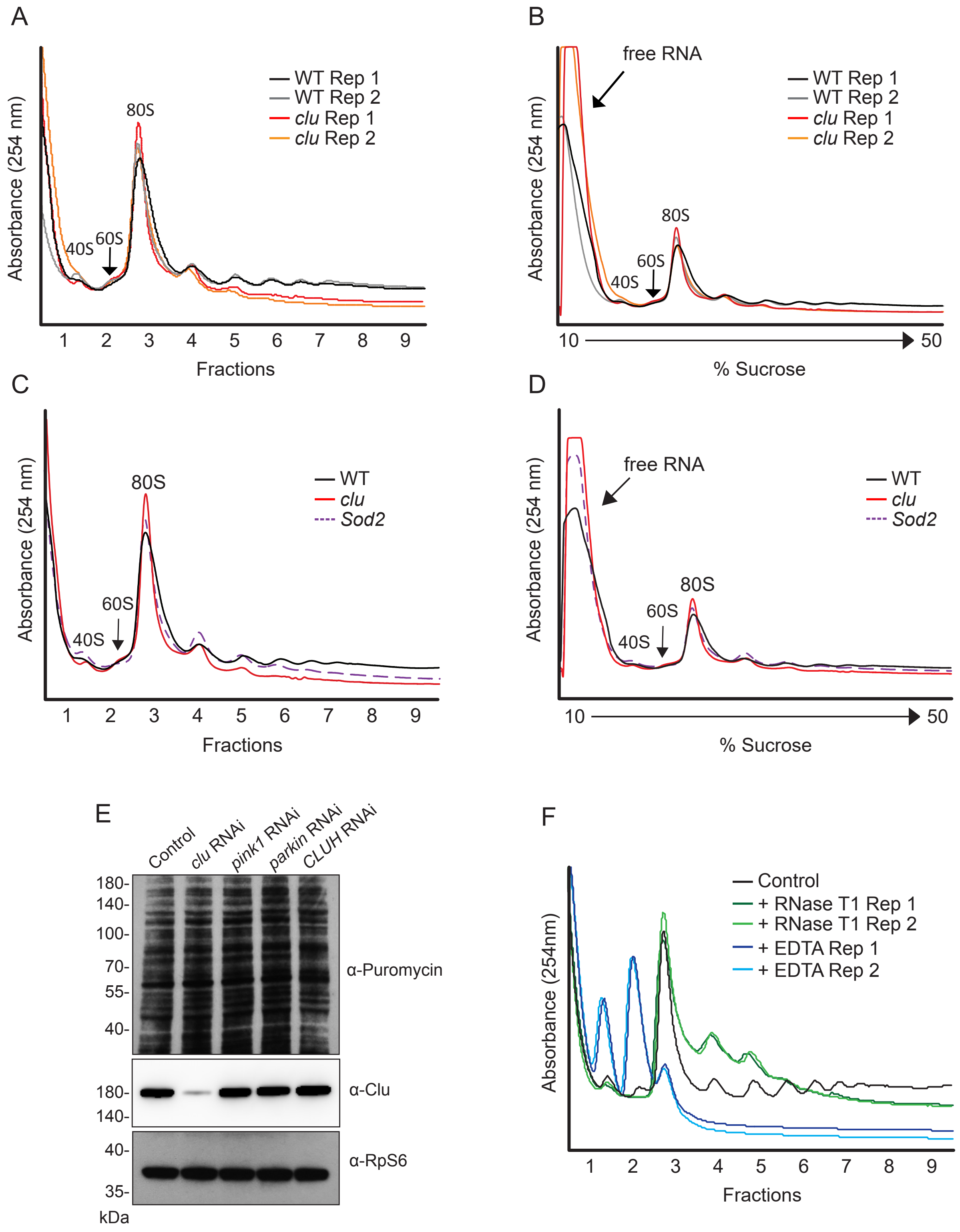

Figure S1 Polysome profile replicates and S2R+ cell puromycin. (A) Polysome profiles of biological replicates for wild type (WT, black/grey traces) and clu mutant adults (red/orange traces). (B) The same polysome profiles from panel A with the trace zoomed out, extending the y-axis to visualize the free RNA peak. (C) Polysome profiles of Superoxide Dismutase 2 (Sod2) mutants (dashed purple trace) compared to (WT, black trace) and clu null mutant (red trace) as shown in (A) for comparison. (D) The same polysome profiles from panel C with the trace zoomed out, extending the y-axis to visualize the free RNA peak. (E) Puromycin treatment of S2R+ cells treated with clu RNAi. pink1, parkin and CLUH RNAi are included as controls. Parkin loss causes mitochondrial dysfunction. (F) Polysome profile for biological replicates for extract from untreated adult flies (control, black) and extract treated with RNase T1 (green lines) and EDTA (blue lines).
