# Supplementary material for: The Drosophila ribonucleoprotein Clueless is required for ribosome biogenesis in vivo
Source: J Biol Chem. 2024 Oct 30;300(12):107946. doi: 10.1016/j.jbc.2024.107946 (PMC11625335; doi:10.1016/j.jbc.2024.107946)
Supplement: Figure S2 [file mmc2.pdf]

Figure S2 Ribosomal proteins associating with Clu

A

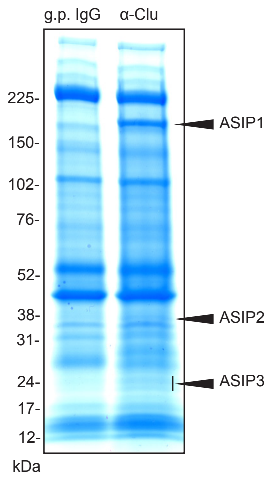

B

RpL	% RA	RpS	% RA
RpL11	19.0	RpS7	50.0
RpL7A	8.4	RpS4	8.9
RpL14	7.3	RpS6	7.3
RpL7	6.4		
RpL24	4.5		
RpL6	4.3		
RpL8	4.3		
RpL10Ab	1.2		

C

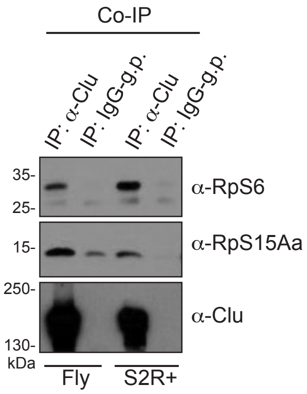

D

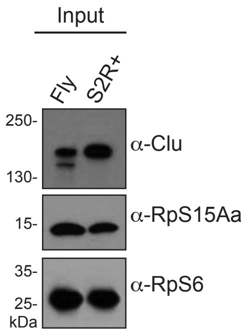

Figure S2 Mass spectrometry analysis of unique Clu-associated bands. (A) Coomassie-stained gel comparing immunoprecipitations from adult extract using α-Clu antibody (right) and guinea pig (g.p.) IgG control (left). The three bands excised for mass spectrometry analysis are indicated by arrowheads. Analysis of the top band (ASIP1) confirmed it is primarily Clu protein. (B) List of ribosomal proteins identified from the lower Clu-unique bands. RpL = Large ribosomal protein, RpS = Small ribosomal protein, RA = Relative abundance. The full list of identified proteins can be found in Table S2. (C) Western blots of RpS6 and RpS15A immunoprecipitations performed from extract isolated from adult flies (Fly) and S2R+ cells using anti-Clu antibodies or control IgG guinea pig (g.p.) antibodies. (D) Input of the extract from Fly and S2R+ cells that was used for the co-IP experiments shown in (C).
