# Supplementary material for: The Drosophila ribonucleoprotein Clueless is required for ribosome biogenesis in vivo
Source: J Biol Chem. 2024 Oct 30;300(12):107946. doi: 10.1016/j.jbc.2024.107946 (PMC11625335; doi:10.1016/j.jbc.2024.107946)
Supplement: Figure S3 [file mmc3.pdf]

Fig. S3 rRNA processing and qPCR primers

A

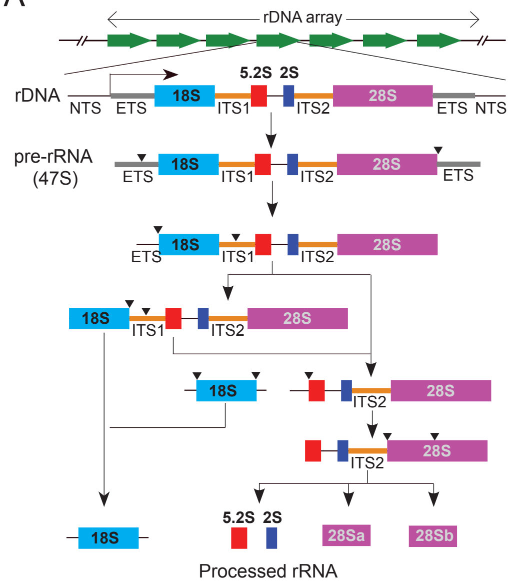

B

qPCR primer locations on cDNA

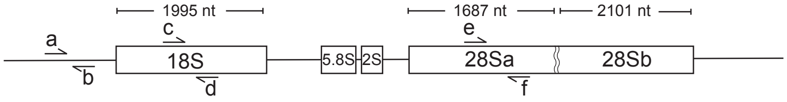

C

qPCR primers

Primer #	Name	Sequence	Target
AS295	a	ATCCGCGGATAATAGGAATATACG	ETS1-For
AS296	b	TAATCCAACTTTACAGGCAGG	ETS1-Rev
AS297	c	GTTCGAAGGCGATCAGATACCG	18S-For
AS298	d	CAACCATACTTCCCCCGGAGCC	18S-Rev
AS299	e	AGGGGAAACCCTGATGGAAGACCG	28S-For
AS300	f	TGCACCAGCTATCCTGAGGGAAAC	28S-Rev
AS303	-	ATGGTGTCTCCCACACCGTGCCC	ACT5C-For
AS304	-	GCTCAGCGGTGGTGGTGAAAGAG	ACT5C-Rev

Figure S3 rRNA analysis. (A) Schematic diagram illustrating the transcription and processing of rRNA. The transcript lengths in the diagram are not to scale. (B) qPCR primer locations in ribosomal cDNA that were used for Figure 4C. (C) Table of primer sequences and their targets that were used for the qPCR analysis in Figure 4C.
