# Supplementary material for: The Drosophila ribonucleoprotein Clueless is required for ribosome biogenesis in vivo
Source: J Biol Chem. 2024 Oct 30;300(12):107946. doi: 10.1016/j.jbc.2024.107946 (PMC11625335; doi:10.1016/j.jbc.2024.107946)
Supplement: Supporting information [file mmc4.docx]

**The Drosophila ribonucleoprotein Clueless is required for ribosome biogenesis in vivo**

Aditya Sen, Ambar Rodriguez-Martinez, Sara K. Young-Baird, Rachel T. Cox

Materials included:

Supporting materials and methods

Figure S1

Figure S2

Figure S3

Figure S4

**Supporting materials and methods**

**Puromycin assay in S2R+ cells**:

Cells were incubated for 60 min in Schneider’s Drosophila Medium (cat # 21720024, Thermo Fisher Scientific, Waltham, MA, United States) containing 10 µg/ml of puromycin (cat # A11138-03, Thermo Fisher Scientific, Waltham, MA, United States). After washing once with 1X Phosphate Buffered Solution (PBS, 137 mM NaCl, 2.7 mM KCL, 10 mM Na_2_HPO_4_, 1.8 mM KH_2_PO_4_), the cells were snap-frozen for further processing.

**S2R+ cell RNAi:**

The S2R+ RNAi method was conducted as previously described (Sen et al., 2015). In brief, cells were cultured in Schneider’s Drosophila Medium (Cat # 21720024, Thermo Fisher Scientific, Waltham, MA, United States) supplemented with 10% fetal bovine serum (CPS Serum, Parkville, MO, USA, cat#. FBS-500HI) and 0.5% Penstrep (Fisher Scientific, Hampton, NH, USA, cat#. BW17602E). Clu dsRNA was synthesized from the 3′ UTR of *clu* using the cDNA clone RH51925 (DGRC Stock 1194281; [https://dgrc.bio.indiana.edu//stock/1194281](https://dgrc.bio.indiana.edu/stock/1194281); RRID: DGRC_1194281) as template and following primers: 5′ TAATACGACTCACTATAGGGAGAACGCTCCCAATGGGCGATG-3′ and 5′-TAATACGACTCACTATAGGGAGACGGACGTGTCTGGTGATCCCG-3′. We used HiScribe^®^ T7 High Yield RNA Synthesis Kit (Cat # E2040S, New England Biolab, Ipswich, MA, United States) to synthesize dsRNA. 1x10^5^ cells were seeded per well in a 48-well plate and the cells were cultured in media containing 25 μM dsRNA for 4 days, with an additional 25 μM dsRNA added on the second day. Subsequently, the cells were processed for western blotting and immunofluorescence as described in the materials and methods. The experiment was carried out at three different time points.

**RT-PCR:**

Total RNAs were isolated from wildtype and mutant adult flies using the Direct-zol RNA Miniprep Plus kit (Cat # R2071, Zymo Research, Irvine, CA). RNA was stored at -80 °C until further use. RT-PCR was performed using NEB one-step RT-PCR kit following the manufacturer’s directions (Cat # E5315S, New England Biolab, Ipswich, MA, United States) and gene specific primers (Figure S4B). Product was run on 1% agarose gels containing appropriate amount of GelRed fluorescent nucleic acid stain (Cat # SCT123, Millipore Sigma, St. Louis, MO). Finally, the gels were visualized under a UV transilluminator.

Sen, A., Kalvakuri, S., Bodmer, R., & Cox, R. T. (2015). Clueless, a protein required for mitochondrial function, interacts with the PINK1-Parkin complex in Drosophila. *DMM Disease Models and Mechanisms*, *8*(6). <https://doi.org/10.1242/dmm.019208>
