# Supplementary material for: The Drosophila ribonucleoprotein Clueless is required for ribosome biogenesis in vivo
Source: J Biol Chem. 2024 Oct 30;300(12):107946. doi: 10.1016/j.jbc.2024.107946 (PMC11625335; doi:10.1016/j.jbc.2024.107946)
Supplement: Figure S4 [file mmc7.pdf]

Fig. S4 Levels rRNA intermediates are not disrupted with Clu loss

A

RT-PCR primer locations on cDNA

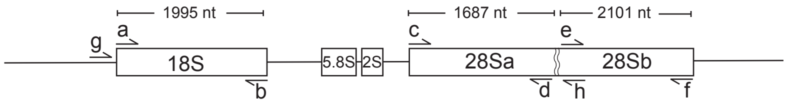

B

RT-PCR primers

Primer #	Name	Sequence	Target
AS315	a	ATTCTGGTTG ATCCTGCCAGTAG	18S-For
AS316	b	TAATGATCCTTCCGCAGGTTCACC	18S-Rev
AS317	c	ACCTCAACTCATATGGGACTACCCCC	28Sa-For
AS319	d	GGCTTTCGCCTTGAACTTAGGACCG	28Sa-Rev
AS320	e	GGGAATACGGTTCCAATTCCG	28Sb-For
AS321	f	CAGTGGATCGCAGTATGGCAGC	28Sb-Rev
AS307	g	AAAAATGGCCGTATTCGAATGG	18S-FJ-For
AS318	h	CTTCCATGGTAGTACGGC	28Sa-EJ-Rev
AS104	-	CACCATGATTGAAATGAACAAAACT-GCAATCGGC	TOM20-For
AS105	-	TTCGAGGTCGTCGATACTTGCG	TOM20-Rev

C

RT-PCR

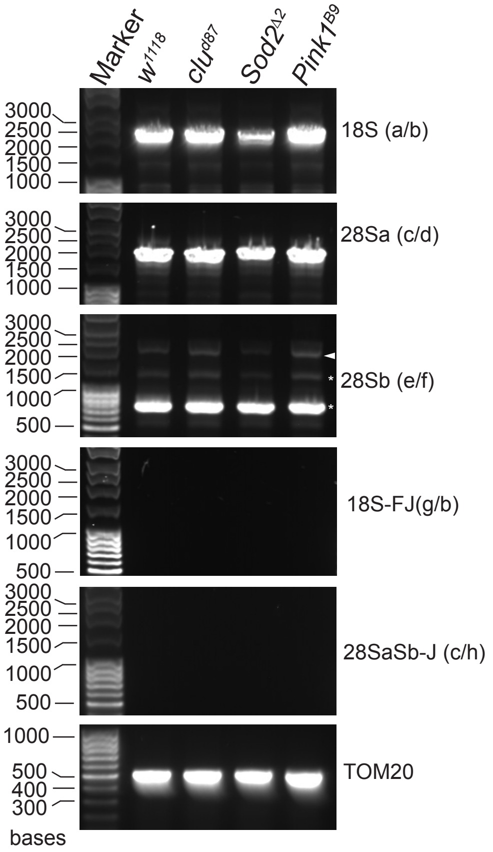

D

qPCR primer locations on cDNA

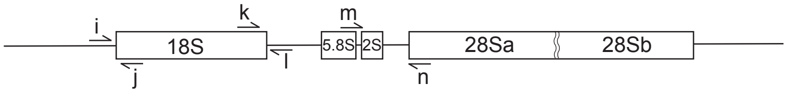

E

qPCR primers and CT-values

Primer #	Name	Sequence	Target	Ave. CT Values ± SD			
				WT	*clu*	*Sod2*	*Pink1*
AS307	i	AAAAATGGCCGTATTCGAATGG	18S-FJ-For	UD	UD	UD	UD
AS308	j	TAACTACTGGCAGGATCAACCAG	18S-FJ-Rev				
AS309	k	GGTTTCCGTAGGTGAACCTGCGG	18S-EJ-For	UD	UD	UD	UD
AS310	l	TGGTGAGGTTGCCAAGCCTCACAC	18S-EJ-Rev				
AS311	m	GAACGCATATCGCAGTCCATGCTG	28Sa-FJ-For	26.04 ± 0.73	27.09 ± 1.4	26.78 ± 2.1	25.32 ± 1.8
AS312	n	GGGGGTAGTCCCATATGAGTTGAGG	28Sa-FJ-Rev				
AS303	-	ATGGTGTCTCCCACACCGTGCCC	ACT5C-For	18.21 ± 0.15	17.36 ± 0.11	19.57 ± 0.04	16.85 ± 0.08
AS304	-	GCTCAGCGGTGGTGGTGAAAGAG	ACT5C-Rev				

Figure S4 Levels of rRNA intermediates are not disrupted with Clu loss. (A) Schematic illustrating the primer location used for RT-PCR analysis in panel (C). (B) Table of primer sequences used for RT-PCR analysis in panel (C). (C) Agarose gels showing the resulting product of each RT-PCR reaction. For 28Sb analysis, the top band is the correct size (arrowhead). Asterisks indicate nonspecific product. (D) Schematic illustrating the primer located used for qPCR analysis in panel (E). (E) Table of primer sequences for qPCR analysis. qPCR for junctions gave no product indicating complete junction cleavage occurs (UD = undetermined). Control primers are for ACT5C. Primers “m” and “n” for 28Sa recognize a late-stage cleavage event that appears to be less transient compared to the other junction primers. CT values are averaged from four technical replicates. SD = standard deviation. For = forward primer, Rev = reverse primer, FJ = front junction, EJ = end junction.
